# Blocking the voltage‐gated sodium channel hNa_v_1.5 as a novel pH‐dependent mechanism of action for tamoxifen

**DOI:** 10.1002/2211-5463.70091

**Published:** 2025-07-31

**Authors:** Karl Josef Föhr, Michael Fauler, Thomas Zimmer, Bettina Jungwirth, Hubert Schrezenmeier, David Alexander Christian Messerer

**Affiliations:** ^1^ Department of Anesthesiology and Intensive Care Medicine University Hospital Ulm Germany; ^2^ Institute of Transfusion Medicine and Immunogenetics Ulm German Red Cross Blood Transfusion Service Baden‐Württemberg‐Hessen and University Hospital Ulm Germany; ^3^ Institute of General Physiology University of Ulm Germany; ^4^ Institute of Physiology University Hospital of Jena Germany; ^5^ Institute of Transfusion Medicine University Hospital Ulm Germany

**Keywords:** breast cancer, patch clamp, selective estrogen receptor modulator, tamoxifen, tumor therapy, voltage‐gated sodium channel

## Abstract

Tamoxifen, a selective estrogen receptor modulator, is widely used in breast cancer treatment, but also affects estrogen receptor‐negative tumors, suggesting alternative mechanisms. Voltage‐gated sodium channels (VGSCs) are implicated in metastasis, making them potential therapeutic targets. However, broad VGSC inhibition is impractical due to their essential physiological roles. Ideally, blockers should selectively target tumor‐associated VGSC properties while sparing normal cells. Since tamoxifen exhibits sodium channel‐blocking activity, we investigated its effects on tumor‐specific VGSC parameters. Electrophysiological experiments using the patch‐clamp technique were conducted on heterologously expressed human cardiac sodium channels (hNa_v_1.5). Tamoxifen does not differentiate between the adult and embryonic splice variants of hNa_v_1.5, the latter being predominant in tumors. However, it effectively blocks hNa_v_1.5 in gating states (slow‐inactivated and open) assumed to be prevalent in cancer cells. Binding affinity increases significantly under acidic conditions (pH 6.0 vs. 7.4), mimicking the tumor microenvironment. The affinity for the slow‐inactivated state was 0.87 ± 0.16 μm at pH 7.4 and 0.16 ± 0.02 μm at pH 6.0. For the open state, half‐maximal inhibition occurred at 2.13 ± 0.08 μm and 0.57 ± 0.02 μm, respectively. Tamoxifen preferentially binds VGSCs under conditions characteristic of cancer tissue, particularly at acidic pH, suggesting its potential as a selective tumor‐targeting agent.

## Graphical abstract

Patch‐clamp recordings revealed that tamoxifen inhibits voltage‐gated sodium channels, especially under acidic conditions, both common in metastatic cancer cells. These effects may explain certain antitumor properties of tamoxifen, highlighting a novel mechanism of action beyond its known endocrine effects.

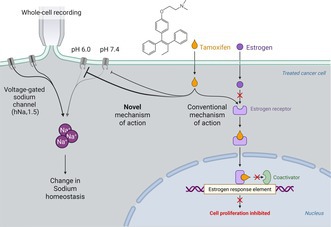

**Abbreviation**
VGSC: voltage‐gated sodium channel

Tamoxifen, a nonsteroidal triphenylethylene derivative, is widely recognized as a selective estrogen receptor modulator. Initially, it was used in the treatment of breast cancer, where it has been prescribed to more than 200 million patients worldwide [1]. Over time, it has also been suggested to be an effective therapy option in various nonbreast, nongynecological carcinomas [2, 3]. This broader range of applications is based on the observation that tamoxifen also affects tumor cells lacking estrogen receptors, suggesting alternative mechanisms of action involving different targets [4]. The list of putative targets is extensive and includes various ligand‐ and voltage‐gated ion channels [5, 6, 7, 8]. For example, tamoxifen can inhibit hERG ion channel function, which may contribute to its anticancer properties [9]. In the context of tumor therapy, voltage‐gated sodium channels (VGSCs) are of particular interest, as they play a key role in the process of metastasis [10, 11]. The fundamental sodium channel‐blocking activity of tamoxifen, with half‐maximal effects observed in the low micromolar range, has been recognized for decades [12]. More recently, a detailed analysis of the mode of action has been conducted, showing that tamoxifen interacts with sodium channels over a slow time scale via an as‐yet‐unknown interaction site [1]. It is worth noting that these findings were derived from investigations of a bacterial sodium channel.

The use of a VGSC blocker in tumor therapy raises several critical considerations. Most importantly, the blocker should selectively target VGSCs in tumor cells while sparing those in healthy cells. In this context, selectively targeting the embryonic isoform versus the adult isoform of the human heart muscle sodium channels (hNa_v_1.5) would be advantageous, as the embryonic isoform is predominant in tumor cells [13]. Additionally, greater potency for the persistent, noninactivating current component compared to the transient current component is desirable, as the hypoxic environment of tumor tissue promotes an increase in persistent current [14, 15]. Furthermore, a drug should preferentially interact with the slow‐inactivated state, as this state is thought to be predominant in tumor cells due to their less polarized resting potential. Finally, increased affinity at low pH is desirable, as tumor cells exist in an acidic environment [16]. The present manuscript investigates the interaction between tamoxifen and VGSCs, addressing these tumor‐specific conditions, with a particular focus on pH‐dependent behavior.

## Materials and methods

### Cell culture

The tsA201 cell line is a transformed human embryonic kidney 293 (HEK293) cell line that stably expresses an SV40 temperature‐sensitive T antigen (Sigma‐Aldrich #85120602; Darmstadt, Germany). By means of an appropriate PCR test, it can be confirmed that all experiments were performed with mycoplasma‐free cells. tsA201 cells were cultured at 37 °C in a humidified atmosphere of 95% air and 5% CO_2_ in minimum essential medium (MEM) supplemented with 50 U·mL^−1^ penicillin, 50 μg·mL^−1^ streptomycin (Gibco, Eggenstein, Germany), 2 mm L‐glutamine (Boehringer, Mannheim, Germany), and 10% fetal bovine serum (Gibco). The cells were grown on polyornithine‐coated culture dishes to 40% confluency and transfected using the jetPei transfection kit (Polyplus, Illkirch, France). The construction of the plasmid pTSV40G‐hNa_v_1.5, encoding wild‐type hNa_v_1.5, has been previously described [17]. This plasmid facilitates the selection of transfected cells, as EGFP is simultaneously produced from a separate expression cassette. Mutant channels (hNa_v_1.5_CW and hNa_v_1.5_F1760K) were obtained by site‐specific modifications of oligonucleotides and overlapping PCR. The PCR fragments were inserted into the pTSV40G‐hNa_v_1.5 background using restriction sites AgeI/BsaBI (for CW) and BstEII/SpeI (for F1760K), resulting in pTSV40G‐hNa_v_1.5_CW and pTSV40G‐hNa_v_1.5_F1760K.

### Electrophysiology

Electrophysiological experiments were performed as described in previous studies [18, 19, 20]. Membrane currents were recorded in the whole‐cell recording mode using an EPC‐9 amplifier and Patchmaster software (v2×73; HEKA, Lambrecht, Germany; [21]). Prior to recording, cells were rinsed twice with an extracellular standard solution containing (in mM): 140 NaCl, 5 KCl, 1.5 CaCl_2_, 2 MgCl_2_, 10 glucose, and 12 HEPES (4‐(2‐hydroxyethyl)piperazine‐1‐ethanesulfonic acid, N‐(2‐hydroxyethyl)piperazine‐N′‐(2‐ethanesulfonic acid); pH 7.3). Patch pipettes were fabricated from borosilicate glass and had tip resistances of approximately 2 MΩ when filled with (in mm): 125 CsF, 10 NaF, 10 EGTA (ethylene glycol‐bis(2‐aminoethylether)‐N,N,N′,N′‐tetraacetic acid), and 10 HEPES; pH 7.2. To improve sealing, the pipette tips were briefly dipped into a 2% solution of dimethylsilane dissolved in dichloromethane.

Unless otherwise stated, the membrane potential was held at −140 mV, from which channel activations were elicited by brief depolarizing pulses to −20 mV with a duration of 5 ms. To minimize voltage errors, series resistance was compensated by up to 80%. Cells with currents exceeding 6 nA were excluded from evaluation. Details of specific protocols are provided in the figure legends where appropriate.

### Drug application

The medium in the dish (1.5 mL) was continuously exchanged via a “global” bath perfusion system, with the inflow set to 4.5 mL·min^−1^, with the outflow removing excess fluid. Reagents were applied locally to the cells using the L/M‐SPS‐8 superfusion system (List, Darmstadt, Germany). Switching between the eight channels of the superfusion system was controlled by magnetic valves. The local inlet (the tip of an eight‐barrel pipette) was positioned 50‐ to 100‐μm upstream, and the local outlet was placed approximately 300‐μm downstream of the patch pipette. A constant flow rate of control and test solutions (1 mL·min^−1^) was maintained using a pressure control system (MPCU‐3; Lorenz, Göttingen, Germany). The solution exchange time was estimated from changes in the liquid junction potential to be approximately 1 ms. Unless otherwise stated, drugs were pre‐applied for 30 s prior to the start of recordings.

### Chemicals

Trypsin was obtained from Biochrom AG (Berlin, Germany). DNase I was obtained from Invitrogen (Carlsbad, Germany), and fetal bovine serum was obtained from HyClone, Perbio Science (Bonn, Germany). Poly‐L‐ornithine was purchased from Sigma‐Aldrich (Schnelldorf, Germany), and tamoxifen was purchased from Tocris (Köln, Germany). All other chemicals were obtained from Sigma‐Aldrich Chemie GmbH (Steinheim, Germany).

### Data analysis and statistics

#### Concentration‐inhibition curves

Concentration‐inhibition curves for the estimation of IC_50_ values were fit to the Hill equation.

(1) $$\frac{I_{D}}{I_{C}}=\frac{1}{1+{\left(\frac{\left[D\right]}{{\mathrm{IC}}_{50}}\right)}^{n}}$$

*I*_*D*_ and *I*_*C*_ represent the current amplitudes in the presence and absence of the drug, respectively, where [*D*] denotes the drug concentration. IC_50_ refers to the concentration of the blocker that results in 50% inhibition, and n represents the Hill coefficient.

#### Voltage‐dependent behavior

The voltage‐dependence of slow inactivation was fit using an extended Boltzmann equation that included an additional parameter (*S*) to account for the steady‐state level of incomplete inactivation.

(2) $$\frac{I}{I_{\max}}=\left(1-S\right)\left(\frac{1}{1+e^{\left(\frac{V-V_{50}}{k}\right)}}\right)+S$$

Abbreviations for voltage‐dependent parameters are as follows: *V* represents the actual clamp potential, and *V*_50_ denotes the potential at which half‐maximal current (*I*) occurs. The slope factor is denoted by *k*.

#### Recovery from inactivation

Current amplitudes were normalized to the maximum peak amplitude of *I*_Na_ both in the absence and presence of tamoxifen. Recovery time constants were estimated using double‐exponential fits, as described by the following equation:

(3) $$\frac{I}{I_{\max}}=1-a_{1}e^{\left(\frac{-t}{\tau_{1}}\right)}-a_{2}*e^{\left(\frac{-t}{\tau_{2}}\right)}$$

Abbreviations used are as follows: *t* represents the time of inactivation, *τ*_1_ denotes individual time constants, and *a*_*i*_ represents the relative contribution of the corresponding terms.

All curve‐fitting procedures were performed using SigmaPlot 13.0 (Sysstat, San Jose, California, USA). Graphical abstract was created using biorender.com. Unless explicitly indicated by error bars, graphs display representative data from single cells. Average values, based on data from at least *n* = 5 cells, are reported as mean ± SD in the results section and figure legends.

## Results

### Tamoxifen blocks human hNa_v_1.5 channels

The principal capability of tamoxifen to block VGSCs has long been recognized from experiments on neuronal cells [12]. Our preliminary experiments confirmed that this capability also extends to hNa_v1_.5 channels, which are primarily expressed in cardiac muscle. However, this manuscript is focused on the question of whether tamoxifen is more effective at blocking VGSCs of tumor cells while sparing those of healthy ones. Given that tumor cells predominantly exist in an acidic environment, we tested the hypothesis that tamoxifen might be more effective at acidic compared to physiological pH. We conducted experiments at pH 7.4 and pH 6.0. Since protons exert a subtype‐specific effect on VGSCs, we first analyzed this aspect in hNa_v_1.5 channels under our experimental conditions.

### General effects of acidification on hNa_v_1.5 currents

**Fig. 1:**
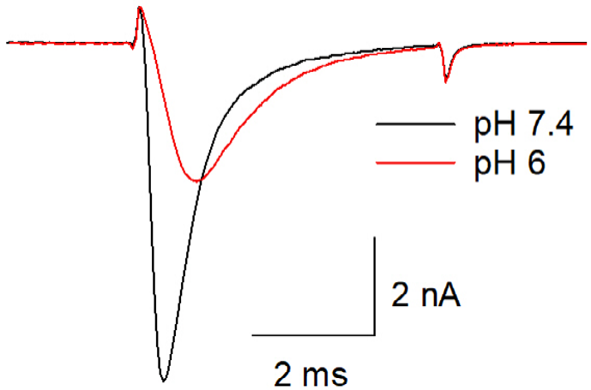
Typical current traces from hNa_v_1.5 channels at pH 7.4 and pH 6.0. Overlay of representative current traces obtained from the activation of hna_v_1.5 channels at ph 7.4 (black) and ph 6.0 (red). of note, kinetics are slowed at ph 6 compared to ph 7.4. current amplitudes at ph 6.0 are reduced to 55.6 ± 6.8% of its size measured at ph 7.4.

When hNa_v_1.5 channels were activated in individual cells at a physiological pH of 7.4 and an acidic pH of 6.0, the currents differed significantly in several aspects (Fig. 1). First, the current amplitudes of resting channels were reduced to 55.6 ± 6.8% of those at pH 7.4 (Fig. 1). Second, the activation and fast inactivation kinetics were slowed at pH 6.0. Third, the proportion of inactivated channels increased from 38.5 ± 6.4% to 62.3 ± 7.9% (taken from the relative current amplitudes obtained for control(inact) vs. control(rest) as illustrated by Fig. 4). All pH‐mediated effects were fully and immediately reversible (data not shown).

### Time‐dependent interaction

Tamoxifen has been reported to interact with a bacterial VGSC on a slow time scale [1]. In order to test this, we employed a protocol appropriate for analyzing a possible interaction with the fast inactivated state. Briefly, channels were inactivated for 500 ms before the test pulse to −20 mV was carried out. It turned out that the endogenous shift of the inactivation curves to more negative potential values was only slightly increased in the presence of tamoxifen. In particular, at pH 7.4, a shift of −2.1 ± 0.9 mV was estimated for 1000 nm, and at pH 6.0, a shift of −0.9 ± 0.9 mV was estimated for 250 nm tamoxifen. These concentrations turned out to be more than half‐maximal effective in other experimental settings (Fig. 4C). Inactivation midpoints for control in these experiments were −78.3 ± 2.2 mV at pH 7.4 and −76.4 ± 2.7 mV at pH 6.0. Altogether, the outcome of these experiments argues at first glance against an interaction with the fast inactivated state.

**Fig. 2:**
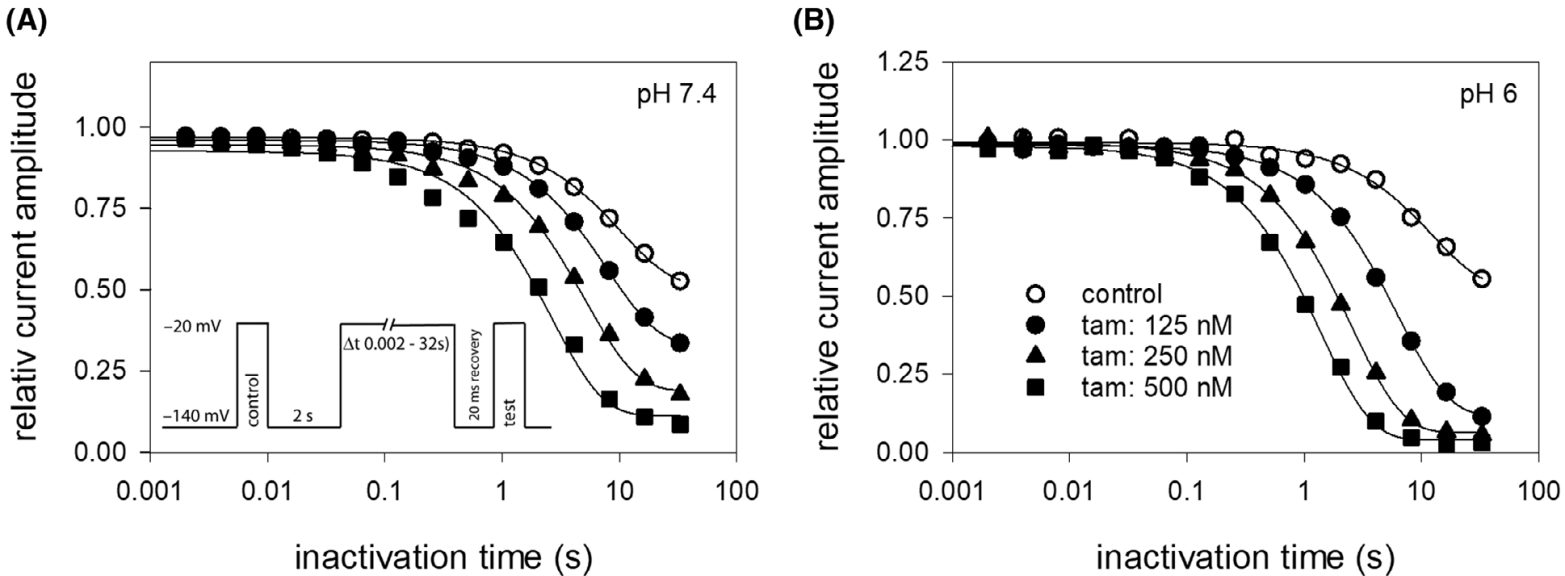
Time‐dependent interaction of tamoxifen with hNa_v_1.5 channels. Illustrated are relative current amplitudes, which were obtained upon channel inactivation for different times at −20 mV. Immediately before the test pulse, a short recovery pulse was inserted (see also insert to Fig. 2A). Data were fit with single exponential functions. It is evident that at pH 7.4, at least at higher concentrations of tamoxifen (filled squares) two exponential functions would be required for an appropriate fit of the data. Tamoxifen caused, at pH 6.0, a stronger decline of channel availability with a smaller residual current compared to pH 7.4 (*n* = 5).

In a next step, we analyzed the time dependency of the interaction with inactivated states on a broader time scale. The protocol is illustrated as inset to Fig. 2A. In brief, following channel activation from a resting potential of −140 mV, sufficient time was allowed for full recovery at the holding potential. The channels were then inactivated for a variable duration (range: 2 ms to 32 s) at a potential of −20 mV. Immediately prior to the test pulse, channels were allowed to recover at −140 mV, thereby eliminating or minimizing the impact from fast inactivation.

Using this protocol in the absence of tamoxifen, significant reductions in availability were observed at inactivation times exceeding 1 s (Fig. 2). At the longest inactivation time (32 s), the remaining current amplitude, compared to that of resting channels, was 56.2 ± 7.6% at pH 7.4 and 54.4 ± 5.4% at pH 6.0. In the presence of tamoxifen, availability decreased at shorter inactivation times, with a far more pronounced reduction at the longest inactivation time. Altogether, these results point to a slow interaction of tamoxifen with the hNa_v_1.5.

A common procedure for obtaining affinity constants from kinetic data involves plotting the inverse of the time constants against the applied drug concentration. If the data points exhibit a linear relationship, *K*_*i*_ values can be calculated using the equation *K*_*i*_ = *k*_off_/*k*_on_. However, this approach could not be applied here, as depending on the pH, either one (pH 6.0) or two exponential functions (pH 7.4) were required to describe the time course of block development (Fig. 2). Thus, at present, these data can only be described qualitatively. For a more accurate evaluation, new analytical procedures have to be established.

### Voltage dependence of slow inactivation

**Fig. 3:**
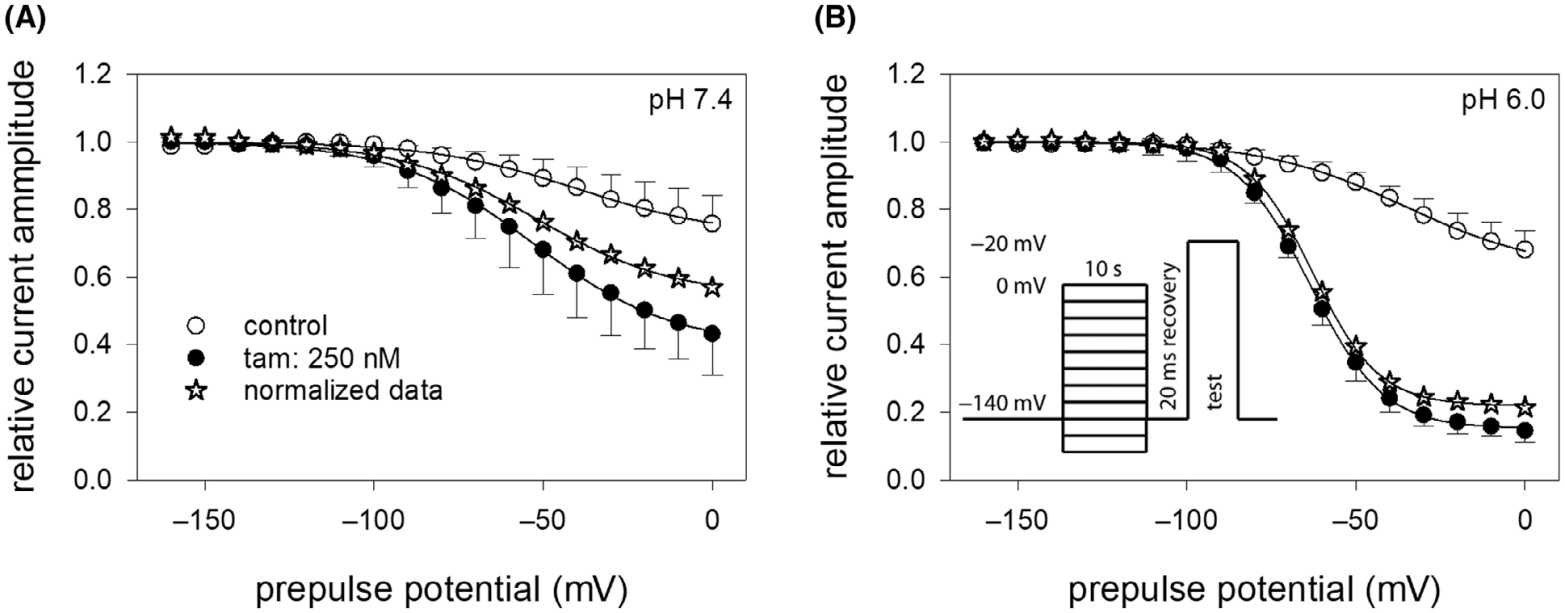
Interaction with the slow‐inactivated state: potential‐dependency. Illustrated are relative current amplitudes in relation to the applied prepulse potentials with experiments carried out at pH 7.4 (A) and pH 6 (B). The experimental scheme is illustrated as inset in (B). After 10‐s prepulses to different potentials (range: −160 to 0 mV), channels were allowed to recover at holding potential (−140 mV) before the test pulse to −20 mV was applied. Current amplitudes are related to the largest amplitude of the individual runs. Data points represent the mean ± SD from 5 independent experiments. Data were fit according to Eqn (2). Half‐maximal inactivation occurred at −39.8 ± 2.8 and − 35.5 ± 1.7 mV for control at pH 7.4 and pH 6.0, respectively. In the presence of tamoxifen (250 nm), half‐maximal inactivation occurred at – 52.8 ± 1.0 and − 63.5 ± 0.3 mV with remaining currents at the prepulse potential of 0 mV of 0.76 ± 0.08 and 0.68 ± 0.06 for control at pH 7.4 and pH 6.0, respectively. The corresponding values in the presence of 250 nm tamoxifen were 0.43 ± 0.12 and 0.15 ± 0.03. Stars indicate normalized data with respect to controls (*n* = 5).

In the next step, we analyzed the voltage dependency of slow inactivation by means of a previously established protocol, as illustrated in the inset of Fig. 3B. Briefly, starting from a resting potential of −140 mV, the conditioning prepulse to different potentials was maintained for 10 s, and test pulses were applied following a brief recovery period (20 ms) at resting potential. Control data at pH 7.4 showed a decline in the availability of channels at inactivation potentials positive to −100 mV. At the most depolarized inactivation potential tested (0 mV), the remaining current was 75.7 ± 8.4% of the maximal current amplitude (Fig. 3A). In the presence of 250 nm tamoxifen, the current decline began in approximately the same voltage range. However, the reduction in current was significantly more pronounced, with a remaining current of 43.1 ± 12.1% at 0 mV (Fig. 3A). Half‐maximal inactivation occurred at −39.8 ± 2.8 mV under control conditions and − 52.8 ± 1.0 mV in the presence of 250 nm tamoxifen.

When identical experiments were performed at pH 6, the effect of tamoxifen was notably stronger (Fig. 3B). The remaining currents for the inactivating prepulse to 0 mV were 67.9 ± 5.6% for control and 14.6 ± 3.3% in the presence of tamoxifen (Fig. 3B). Half‐maximal inactivation occurred at −35.5 ± 1.7 mV and − 63.5 ± 0.3 mV under control conditions and with tamoxifen, respectively. As the current amplitude evidently dropped in two steps with the increase in membrane potential, we normalized these data to their corresponding controls (stars in Fig. 3) in order to obtain additional information with which state (fast, slow) the interaction takes place [19, 22]. At pH 6.0, the response clearly followed a biphasic pattern; this was not the case for pH 7.4. In any case, residual normalized current amplitudes continuously decreased with increasing membrane potential.

### Concentration‐dependent interaction: Estimation from steady‐state slow inactivation residual currents

**Fig. 4:**
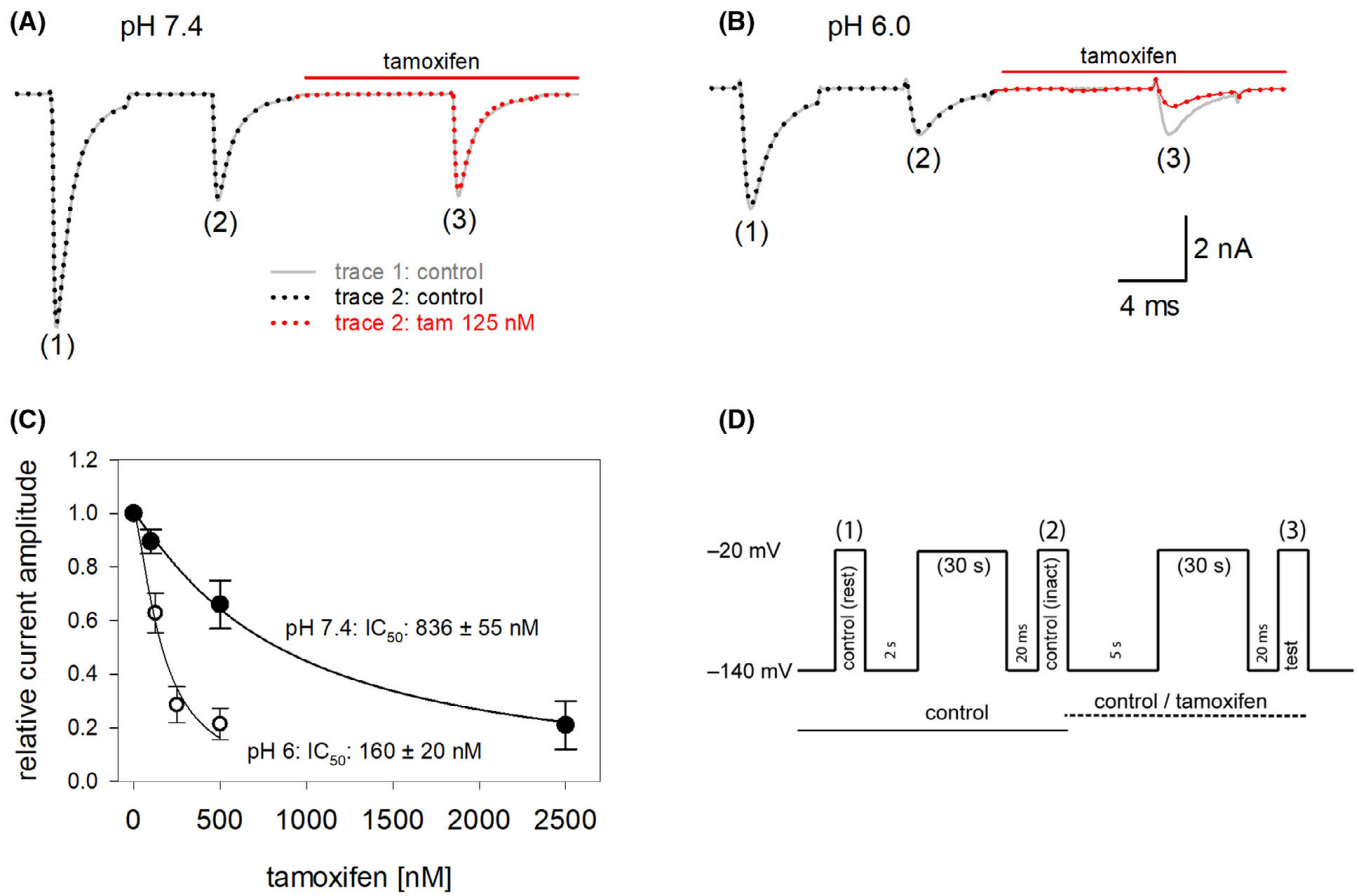
Interaction of tamoxifen with the slow‐inactivated state at pH 7.4 and pH 6.0: Concentration‐dependency. (A, B) Illustrated are original traces obtained from channel activations as shown for the experimental scheme in (D). The three signals represent the current amplitudes for the resting state (1: control rest), inactivated state for control (2: control(inact)) and the inactivated state in the absence or presence of tamoxifen (3: test) at pH 7.4 and pH 6.0. The graphs show the overlay of two traces: one for control currents (gray, continuous line) and a second dotted line, where control is in black and the presence of 125 nm tamoxifen is in red. Bars indicate the application time for tamoxifen for the second trace. All traces (pH 7.4 and pH 6.0) are from the same cell. Longlasting phases at rest (−140 mV) or during inactivation (−20 mV, 30 s) are omitted. (C) Summary of relative current amplitudes from 5 independent experiments are shown as mean ± SD. For evaluation the current amplitudes obtained for test pulses (3) were related to those obtained for control (inact, 2). Data were fit according Eqn (1), resulting in affinities of 836 ± 55 nm and 160 ± 20 nm (*p* < 0.01, unpaired t test) for pH 7.4 and pH 6.0, respectively (*n* = 5).

Given tamoxifen's pronounced interaction with the slow‐inactivated state, we developed a new experimental design to analyze this aspect in greater detail. The corresponding triple‐pulse protocol is depicted in Fig. 4D. The essential part of the protocol consists of two identical sequences executed consecutively in the absence and presence of tamoxifen. In brief, after an initial activation (1: control, rest), sufficient time was provided for full recovery. Thereafter the control part, consisting of a prolonged inactivation (30 s at −20 mV), a short recovery for 20 ms at resting potential, and the activation under control (2: control, inact) was executed. After a second prolonged recovery at resting potential for 5 s, the essential part of the protocol (prolonged inactivation, short recovery) was repeated either under control conditions or in the presence of different concentrations of tamoxifen before the last activation was applied (3: test). Original current traces from the same cell at pH 7.4 and pH 6 are shown in Fig. 4A,B, respectively. Current traces recorded under control conditions are shown in black, while those obtained in the presence of tamoxifen are depicted in red.

To evaluate drug effects, the current amplitudes recorded for test pulses (3) were normalized to those obtained for the corresponding control conditions (2: control, inact). Evaluation was restricted to data in which the current amplitudes obtained for control (control, rest and test) did not deviate by more than 5%.

From five independent experiments performed at pH 7.4 and pH 6.0, we calculated half‐maximal inhibition values of 836 ± 55 nm and 160 ± 20 nm tamoxifen, respectively, with Hill coefficients of 1.15 and 1.47 (Fig. 4C). These results indicate that the affinity of tamoxifen under these experimental conditions is approximately five times higher at pH 6.0 than at pH 7.4.

### Recovery from slow inactivation

**Fig. 5:**
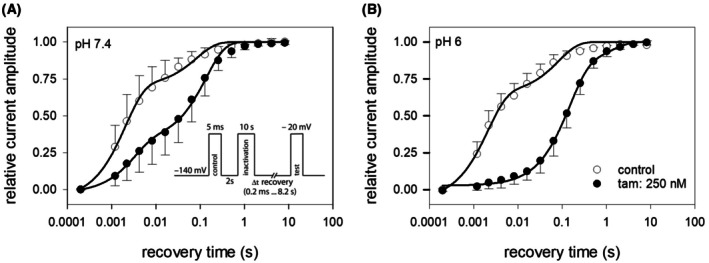
Recovery from slow inactivation. Relative current amplitudes versus the recovery time at −140 mV are illustrated for experiments carried out at pH 7.4 (A) and pH 6.0 (B). The experimental procedure is illustrated as inset to (A). After a control activation, channels were inactivated for 10 s at −20 mV, thereafter they were allowed to recover for a variable duration at −140 mV before the test pulse was carried out. Data points from 5 independent experiments are shown as mean ± SD. Solid lines represent fits of functions with two exponentials (Eqn 3). Current amplitudes of test pulses were normalized to their controls. Interval between individual runs was 10 s and tamoxifen concentration was 250 nm (*n* = 5).

To analyze recovery from inactivation, we employed the pulse protocol as illustrated by the inset to Fig. 5A. The protocol consisted of a control pulse, followed by sufficient time for full recovery. Subsequently, the channels were inactivated for 10 s at −20 mV. Test pulses were applied after the channels were allowed to recover at a holding potential of −140 mV for variable durations (range: 0.2 ms to 8.2 s). For evaluation, the current amplitudes of the test pulses were normalized to their controls and plotted against the recovery time (Fig. 5A). The recovery time course was described by fitting the data points with double‐exponential functions (Eqn 3).

Under control conditions at pH 7.4, the majority of channels (71.9 ± 3.2%) recovered with a time constant of *τ*_1_ = 2.0 ± 0.2 ms, while the remaining channels recovered with *τ*_2_ = 82.9 ± 15.7 ms (Fig. 5A). In the presence of 250 nM tamoxifen, the proportion of fast‐recovering channels decreased to 35.1 ± 2.9%, and both recovery time constants were slightly increased. Specifically, *τ*_1_ and *τ*_2_ were estimated to be 3.1 ± 0.6 ms and 134.7 ± 11.0 ms, respectively.

Control values at pH 6.0 were comparable to those at pH 7.4. Approximately 67.2% of the channels recovered with a time constant of *τ*_1_ = 2.1 ± 0.3 ms, while the remaining channels recovered with *τ*_2_ = 83.7 ± 17.1 ms. However, in the presence of 250 nm tamoxifen, a fast time constant in the low‐millisecond range was no longer observed (Fig. 5B). Instead of this, the fastest time constant τ_1_ was estimated to be 142.7 ± 11.6 ms, accounting for 82.4 ± 4.8% of the channels. The remainder recovered with a time constant of 1.2 ± 0.4 s.

### Interaction with the embryonic subtype of the hNa_v_1.5

Several isoforms of the hNa_v_1.5 channel exist, with the embryonic form being the most significant for tumor cells. Although the embryonic and adult forms differ by the substitution of only seven amino acids, they vary in several electrophysiological parameters [13]. However, aside from a monoclonal antibody, there is no effective pharmacological tool that preferentially targets the embryonic form [23].

**Fig. 6:**
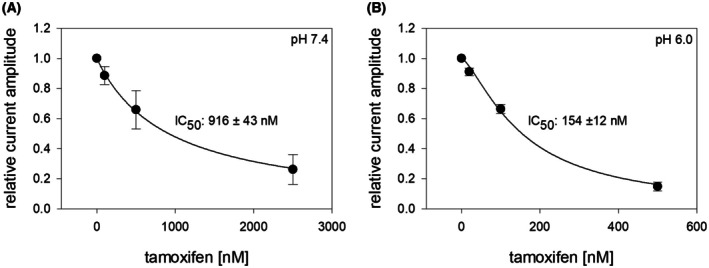
Interaction of tamoxifen with the embryonic hNa_v_1.5 channel. The graph illustrates concentration–response curves obtained from the application of tamoxifen to embryonic hNa_v_1.5 channels at pH 7.4 (A) and pH 6.0 (B). Experimental protocol as shown in Fig. 4. The affinity increases about sixfold when the pH of the extracellular fluid is lowered from pH 7.4 to pH 6.0 (*n* = 5). Data points from 5 independent experiments are shown as mean ± SD.

Nonetheless, we tested tamoxifen for a possible selective activity using our standard test pulse program (Fig. 4D). The results revealed that tamoxifen's affinity for the embryonic form was identical to that observed for the adult form. Specifically, at pH 7.4, an IC_50_ of 916 ± 43 nm was obtained, compared to 154 ± 12 nm at pH 6.0 (Fig. 6). Overall, these values were not significantly different from those measured for the adult channel variant (*P* = 0.42 for pH 6.0 and *P* = 0.82 for pH 7.4, respectively, unpaired *t*‐test). Therefore, tamoxifen does not distinguish between the embryonic and adult isoforms.

### Interaction with the persistent current

**Fig. 7:**
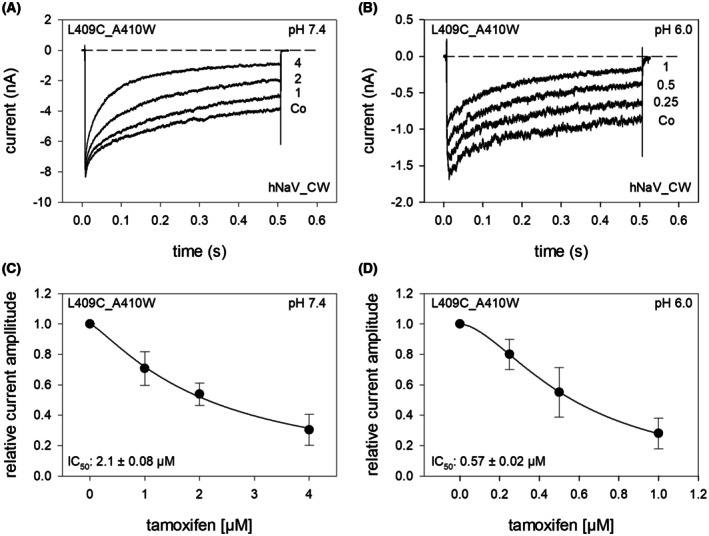
Interaction of tamoxifen with the persistent current mutant hNa_v_1.5 CW. (A, B) The graphs illustrate original current traces obtained from the inactivation deficient mutant upon long‐lasting activations (500 ms) in the absence and presence of different concentrations of tamoxifen at pH 7.4 and pH 6.0. Data from A and B are from different cells. (C, D) Concentration‐response curves were established from the relative current amplitudes obtained immediately before the end of the activation pulses. Half‐maximal inhibition occurred at 2.13 ± 0.08 and 0.57 ± 0.02 μm for pH 7.4 and pH 6.0, respectively. Data points from 5 independent experiments are shown as mean ± SD.

In the next experiments, we evaluated the effect of tamoxifen on the persistent current using a channel mutant with a reduced capacity for inactivation (hNa_v_1.5_L409C_A410W; CW mutant). In this mutant, channels remain largely open even after prolonged activation (500 ms) (Fig. 7). The current amplitudes measured at the end of the activation pulses in the presence of tamoxifen were normalized to those obtained in control solution and plotted as a function of tamoxifen concentration. To estimate the half‐maximal effective concentration (IC_50_), the data were fit with the Hill equation (Eqn 1).

At pH 7.4, the half‐maximal inhibition (IC_50_) was estimated to be 2.13 ± 0.08 μm with a Hill coefficient of *n* = 1.2 ± 0.09. The corresponding values at pH 6.0 were 0.57 ± 0.03 μm with a Hill coefficient of *n* = 1.68 ± 0.01 (Fig. 7). These results indicate that the affinity for tamoxifen increased approximately fivefold when the pH was reduced from 7.4 to 6.0.

### Interaction with the local anesthetic binding site

The binding site for local anesthetics within the channel pore is one of the most prominent interaction sites through which a wide variety of drugs interact with voltage‐gated sodium channels. In the case of tamoxifen, a recent study identified a previously unknown interaction site using investigations of a bacterial sodium channel [1]. To obtain an initial indication of whether this might also apply to the human hNa_v_1.5, we employed the F1760K mutant, one of several mutants known to affect the local anesthetic binding site. If the action of tamoxifen were strongly affected by the F1760K mutant, an interaction with abovementioned new interaction site would be unlikely.

**Fig. 8:**
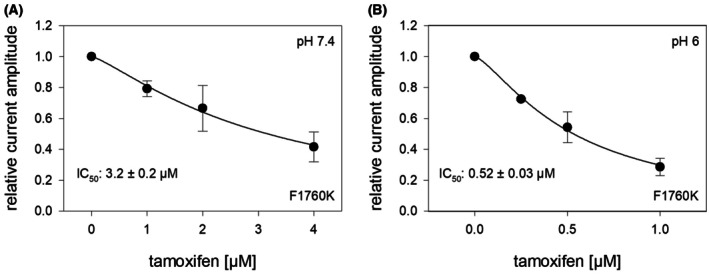
Analysis for a potential interaction of tamoxifen with the local anesthetic binding site. The graph illustrates concentration–response curves for tamoxifen for (A) pH 7.4 and (B) pH 6.0 at the F1760K mutant to test for a possible interaction of tamoxifen with the local anesthetic binding site. Using the experimental procedure as outlined by Fig. 4D, it is shown that the affinity for tamoxifen was only moderately reduced when compared to wild‐type channels (c.f. Fig. 4) Data points from 5 independent experiments are shown as mean ± SD.

Since tamoxifen primarily affects slow processes, we analyzed the mutant channels using the protocol illustrated in Fig. 4D. Overall, the IC_50_ values for tamoxifen did not differ substantially from those of wild‐type channels (0.84 ± 0.06 μm at pH 7.4 and 0.16 ± 0.02 μm at pH 6.0). Specifically, for the mutant channels (F1760K), the IC_50_ at pH 7.4 was 3.2 ± 0.2 μm, and at pH 6.0, it was 0.52 ± 0.03 μm (Fig. 8). Thus, the affinities for tamoxifen at the mutant channels were reduced only three‐ to fourfold at both pH 7.4 and pH 6.0 when compared to the values obtained for wild‐type channels.

## Discussion

The most significant finding of our investigation is the identification of a novel mechanism of action for tamoxifen. Specifically, we discovered that tamoxifen blocks the VGSC hNa_v_1.5 with greater affinity at pH 6.0 than at pH 7.4, which may contribute to its antitumor properties. Furthermore, this characteristic places tamoxifen in a rare class of drugs that exhibit acidotropic behavior [24].

### Voltage‐gated sodium channels and tumor cells

The functional expression of voltage‐gated sodium channels (VGSCs) in tumor cells plays a critical role in acidifying their environment, which enhances proteolytic activity necessary to promote metastasis [10, 25]. Therefore, in the context of therapy, it is crucial to selectively block sodium influx through VGSCs in tumor cells while sparing those in healthy cells.

Within the tumor microenvironment, VGSCs are subject to unique conditions, making specific drug‐receptor (ion channel) interactions particularly relevant for tumor therapy. Notably, tumor cells exhibit a relatively depolarized membrane potential, which causes VGSCs to predominantly reside in various slow‐inactivated states [26]. A subset of channels remains in the open state due to incomplete inactivation, leading to a persistent current. This current may be further enhanced by the acidic environment and reduced oxygen pressure commonly observed in tumor tissues [25]. As a surrogate for the persistent current, we made use of the CW mutant that displays a reduced capacity for inactivation. Here, it should be mentioned that the conformation that causes the persistent current might be different from the conformation that is generated under hypoxic or acidic conditions. Furthermore, this mutation at the intracellular end of the S6 segment in domain I is close to the newly described binding site for tamoxifen, whereby a direct impact of the mutation on the putative binding site cannot be excluded [1, 27]. Altogether, the efficacy for tamoxifen to block the CW mutant of the hNa_v_1.5 channel was less compared to that obtained after a prolonged inactivation.

As such, drugs that selectively target the slow‐inactivated state, suppress persistent current, or exhibit greater efficacy at low pH are of significant therapeutic interest. Importantly, this interest is not limited to tumor therapy, as similar conditions are observed in other pathologies [28]. Another key feature of tumor cells is their preferential expression of the neonatal or embryonic form of VGSCs, which is typically replaced by the adult form during development [13]. Thus, a drug that selectively interacts with the embryonic form of VGSCs is also highly desirable.

### Conditions of extracellular acidification

The proton activity (pH) of blood and tissue is tightly regulated by various mechanisms to maintain an extracellular fluid pH close to 7.4 [29]. However, under pathological conditions such as hypoxia, ischemia, or drug abuse, this value can decrease, approaching a pH of 6.0 [30]. A similarly low pH is also observed in the microenvironment of growing tumors [16]. From a pharmacological perspective, such changes in pH may influence the efficacy of drug–receptor interactions, either by affecting the drug, the receptor, or both.

### General effects of acidic pH on voltage‐gated sodium channels

Extracellular acidification independently modulates the various subtypes of VGSCs in distinct ways. For example, Na_v_1.4 channels in skeletal muscle are minimally affected, whereas Na_v_1.2 and hNa_v_1.5 exhibit opposing effects depending on the protocol used [29]. A common observation, however, is that the current amplitude of all sodium channel subtypes decreases with a reduction in pH, with the most pronounced effect observed in hNa_v_1.5 channels [29]. This phenomenon is generally attributed to protonation of carboxylates in the outer vestibule caused by acidification [31].

Regarding channel characteristics, a decrease in pH results in a shift of the activation and fast inactivation curves to more positive potentials and a slowing of kinetic parameters [29, 32, 33]. In contrast, slow inactivation parameters appear to remain unaffected by changes in extracellular pH from 7.4 to 6.0 [32].

### Electrophysiological implications of the interaction of tamoxifen and hNa_v_1.5 channels

Consistent with previous studies, we observed a reduction in the peak current amplitude of hNa_v_1.5 channels (holding potential: −140 mV) to approximately 55% of its original value when the pH was lowered from 7.4 to 6.0 [29]. In the more physiological potential range, the most notable effects were observed in slow inactivation parameters. In the absence of tamoxifen, the potential‐dependent behavior was similar at both pH values. However, in the presence of tamoxifen (250 nm), the shift in the slow inactivation curve was particularly pronounced at pH 6.0. From the concentration–response relationship, a fivefold increase in efficacy was observed upon switching to the acidic environment (Fig. 4). Even though the strongest effects are observed when using protocols that analyze the interaction with the slow‐inactivated state, this does not necessarily imply that the interaction primarily occurs via this state [19]. Briefly, it is impossible to distinguish an interaction with the slow inactivation state from that obtained due to a slow interaction with the fast inactivation state [34]. If the potential‐dependent behavior is taken as an important criterion considering that slow inactivation develops at more positive membrane potentials than fast inactivation, one could come to the conclusion that at pH 7.4, both fast and slow inactivation are equally important [22, 35]. In the case of pH 6.0, the interaction with the fast inactivated state appears to predominate (the potential‐dependent course of normalized data is given by stars in Fig. 4). Nevertheless, the interaction requires that sodium channels remain in an inactivated state for a prolonged period, a condition that is certainly provided by tumor cells. To balance experimental feasibility with taking this effect into account, we set the inactivation time to 30 s, which we considered to be sufficient from preliminary experiments to reach near equilibrium conditions for all concentrations tested. The half‐maximal effective concentrations estimated here are, at least under acidic conditions, well within the range found in the plasma of treated patients [36, 37]. To the best of our knowledge, no other voltage‐gated sodium channel blocker exhibits several‐fold greater efficacy at acidic compared to physiological pH. In a study evaluating 30 clinically relevant sodium channel blockers, none displayed such a behavior [24]. In contrast, most compounds were less potent at acidic pH than at physiological pH. This discrepancy has been partly attributed to their acidic dissociation constant (pKa), particularly when the pKa is in the neutral pH range. In such cases, the protonation status of the drug varies significantly with pH, altering the proportion of the charged versus uncharged forms of the compound. A notable example is ranolazine, which has a pKa around 7.2. When the pH shifts from 7.4 to 6.0, the proportion of the protonated form increases by more than 60% [33]. This additional charge renders the drug more hydrophilic, potentially impeding its access to the binding site. In contrast, tamoxifen, with a pKa around 8.7, behaves differently. Acidification minimally affects its protonation status, with only about a 4% increase in the charged form. Thus, it is more likely that the acid‐induced increase in tamoxifen's drug affinity is attributable to other factors.

A recent report identified two previously unknown drug interaction sites for tamoxifen at voltage‐gated sodium channels, located near the intracellular gate and the exit of the transmembrane ion‐conducting pore [1]. These findings are based on crystallographic data from a prokaryotic VGSC (*Magnetococcus marinus*), which shares many properties with its eukaryotic counterparts, including drug potency and mechanisms of drug inhibition [38]. Currently, no receptor mutants are known to affect interactions with these newly described binding sites.

We investigated whether tamoxifen interacts with the well‐characterized anesthetic binding site, which is targeted by many other inhibitors of VGSCs [39]. To test this, we utilized the channel mutant F1760K, which significantly alters interactions with this site. For example, in the case of lidocaine—a prototypical local anesthetic—affinity is reduced approximately 25‐fold, whereas for the classical open‐channel blocker flecainide, the reduction is only two‐ to threefold [39].

In our experiments, tamoxifen's affinity was reduced by approximately three‐ to fourfold in the presence of the F1760K mutation. This suggests that the local anesthetic binding site does not play a major role in tamoxifen's mechanism of action. These findings align with those of Sula and coworkers, who proposed that tamoxifen operates via a different, likely newly described, interaction site. Interestingly, the binding pocket at VGSCs appears to resemble the binding pocket at tamoxifen's primary target, the estrogen receptor [1]. Taken together, this raises the intriguing possibility that tamoxifen's interaction with this novel site may underlie its increased affinity at acidic pH. If true, it would be valuable to investigate whether other drugs that do not interact with the local anesthetic binding site also exhibit pH‐dependent changes in their affinity.

### Tumor cells and embryonic VGSCs

Selective targeting of the embryonic form of VGSCs, which is replaced by the adult form in the early postnatal period, appears to be an ideal therapeutic strategy. In the case of hNa_v_1.5, the embryonic and adult forms differ by only seven amino acids, making pharmacological discrimination particularly challenging [13]. To date, an antibody has been shown to block the neonatal hNa_v_1.5 with approximately 400‐fold higher affinity than the adult hNa_v_1.5 [23]. Among small molecules, two well‐known toxins also exhibit differential efficacy; however, they are more effective against the adult form than the embryonic form.

Our investigations indicate that tamoxifen is not suitable for discriminating between the embryonic and adult forms of hNa_v_1.5 channels. Moreover, we were unable to confirm previous reports suggesting greater resistance of the embryonic hNa_v_1.5 to acidification [16]. At other VGSC subtypes, discrimination between the embryonic and adult forms would be even more challenging, as the differences in these subtypes are less pronounced than in hNa_v_1.5 channels [40]. In conclusion, while selective targeting of neonatal hNa_v_1.5 remains a promising potential add‐on therapy for metastatic breast cancer, this strategy cannot be easily extended to other VGSC subtypes [13].

### Clinical implications

The present study was conducted in a cell line not related to gynecologic tumors, which overexpressed hNa_v_1.5 channels. Because hNa_v_1.5 is also expressed in metastatic breast cancer, the interaction of tamoxifen and hNa_v_1.5 might have further potential clinical impact [41, 42]. hNa_v_1.5 has been reported to be involved in the formation of invadopodia, actin‐rich protrusions that facilitate the invasion of tumor cells. Inhibiting hNa_v_1.5 impairs invadopodia formation, thus reducing the cell's invasive capabilities [43]. Moreover, hNa_v_1.5 facilitates extracellular matrix (ECM) degradation by promoting pericellular acidification, enabling cancer cells to invade surrounding tissues. Tamoxifen's blockade of hNa_v_1.5 might therefore reduce ECM degradation, thereby potentially decreasing the invasiveness of invadopodia [43]. Additionally, hNa_v_1.5 colocalized with the sodium‐proton exchanger 1 (NHE‐1) in invadopodia of a breast cancer cell line, allosterically enhancing NHE‐1 activity and contributing to extracellular acidification. Blocking hNa_v_1.5 might disrupt this interaction, reducing NHE‐1 activity and limiting tumor cell invasiveness [43]. In line with this, silencing hNa_v_1.5 expression in breast cancer cells significantly reduces lung colonization in immunodeficient mice, indicating that hNa_v_1.5 is crucial for metastasis [44]. Tamoxifen's blockade of hNa_v_1.5 could similarly suppress metastatic spread. As outlook, the present findings should be confirmed in breast cancer cell lines *in vitro* and in corresponding clinical organoids and/or experimental models of breast cancer.

## Conclusion

Tamoxifen fulfills key criteria for a VGSC blocker that is more effective in cancer cells than in healthy cells. Notably, it demonstrates high affinity for the slow‐inactivated state and increased efficacy in acidic environments. These conditions are rarely encountered in healthy tissue under physiological conditions. Tamoxifen's ability to affect carcinoma cells lacking estrogen receptors may, in part, be attributed to its capability to block VGSCs.

## Conflicts of interest

The authors declare no conflict of interest.

## Author contributions

KJF, DACM contributed to conceptualization. KJF, DACM contribured to data curation. KJF, MF, DACM contributed to formal analysis. DACM contributed to funding acquisition. KJF, MF, DACM contributed to investigation. KJF, MF, TZ contributed to methodology. KJF, TZ; BJ, HS, DACM contributed to resources. KJF, DACM contributed to visualization. DACM, KJF contributed to writing – original draft preparation. All authors contributed to writing – review and editing.

## Data Availability

The data that support the findings of this study are available from the corresponding author david.messerer@uni-ulm.de upon reasonable request.
